# Effects of Tactile Sensitivity on Structural Variability of Digit Forces during Stable Precision Grip

**DOI:** 10.1155/2016/8314561

**Published:** 2016-10-25

**Authors:** Ke Li, Na Wei, Shouwei Yue

**Affiliations:** ^1^Laboratory of Motor Control and Rehabilitation, Institute of Biomedical Engineering, School of Control Science and Engineering, Shandong University, Jinan, China; ^2^Department of Geriatrics, Qilu Hospital, Shandong University, Jinan, China; ^3^Department of Physical Medicine and Rehabilitation, Qilu Hospital, Shandong University, Jinan, China

## Abstract

This study investigated the effects of fingertip tactile sensitivity on the structural variability of thumb and index finger forces during stable precision grip. Thirty right-handed healthy subjects participated in the experiment. Transient perturbation of tactile afferents was achieved by wrapping up the distal pads of the thumb or index finger with transparent polyethylene films. The time-dependent structure of each digit force and the variability of interdigit force correlation were examined by detrended fluctuation analysis (DFA) and detrended cross-correlation analysis (DCCA), respectively. Results showed that the tactile sensitivity affected α_DFA_ of the vertical shear force *Fx* (*F*_3,239_ = 6.814, *p* < 0.001) and α_DCCA_ of *Fx* (*χ* ^2^ = 16.440, *p* < 0.001). No significant difference was observed in α_DFA_ or α_DCCA_ of the normal forces produced by the thumb or index finger. These results suggested that with blurred tactile sensory inputs the central nervous system might decrease the vertical shear force flexibility and increase the interdigit shear force coupling in order to guarantee a stable grip control of an object against gravity. This study shed light on the feedback and feed-forward strategies involved in digit force control and the role of SA-II afferent fibers in regulation of vertical shear force variability for precision grip.

## 1. Introduction

Decades of neuroscience studies have uncovered the close ties between fingertip tactile sensation and digit forces in manipulation tasks. On one hand, fingertip tactile sensation needs to be evoked by a certain amount of force. Information about the amplitude, direction, fluctuation, and spatial distribution of digit forces can be encoded by the cutaneous mechanoreceptors innervating the glabrous skin and further conveyed to the central nervous system (CNS) via afferent fibers [1, 2]. On the other hand, the tactile sensation provides valuable information to adjust the digit forces to suit manipulative actions. In order to generate an appropriate digit force, muscles should be activated by proper motor commands in accordance with the task or environmental variables reflected by tactile information [3, 4]. The tactile-force interaction forms a fundamental but complex mechanism for hand sensorimotor integration [5]. Loss of tactile information following simulated or pathologic sensory lesions may disturb digit forces, leading to difficulties with manual tasks [6–8].

Studies of the tactile-force interaction in manipulation have mostly focused on the dynamic action phases when reaching, lifting, or releasing an object, but relatively little attention was addressed on the static action phase during stably gripping and holding an object. Recently, more and more studies have suggested that the fluctuating digit force in the process of sustained isometric pinch or stable precision grip contains dynamic patterns that might be highly sensitive to sensory modalities. For example, under nonvision condition when only the tactile and proprioceptive information is available, there are lower variability in the time-dependent structure of individual digit force and stronger interdigit force correlation, compared to the condition with visual feedback [9–11]; following a transient median nerve block at the wrist, the impaired sensorimotor system can alter the dynamical force coordination of the thumb and index finger and the structural variability of digit forces [12]. These findings provide evidence that digit force fluctuation is subject to sensorimotor control, yet more studies are needed to examine the role of tactile perception in this process.

The aim of this study was to investigate the effects of tactile sensitivity on the structural variability of digit forces when the thumb cooperates with the index finger for stable precision grip. By experimentally induced perturbation, the fingertip tactile perception could be separately disturbed at each digit, rendering both balanced and unbalanced tactile sensitivity across digits. Structural variability of both the shear (in the vertical or horizontal directions) and the normal (in the perpendicular direction to the contact surface) forces was quantified using dynamical analyses. It was hypothesized that the compromised tactile sensitivity would lower the structural variability of both shear and normal forces at the engaged digits and augment the interdigit force correlation during stable precision grip.

## 2. Methods

### 2.1. Subjects

Thirty healthy right-handed subjects (15 males and 15 females; age = 22.5 ± 1.2 y; height = 168.4 ± 9.5 cm; weight = 61.4 ± 9.6 kg) participated in this study. All participants were senior undergraduate students from the School of Control Science and Engineering, Shandong University. No one reported any history of musculoskeletal or neurological disorders. Each subject was informed and provided with written consent prior to the experiment. This study was approved by the Institutional Review Board of Shandong University and was in accordance with the 1964 Helsinki declaration and its later amendments or comparable ethical standards.

### 2.2. Experimental Setup

**Figure 1:**
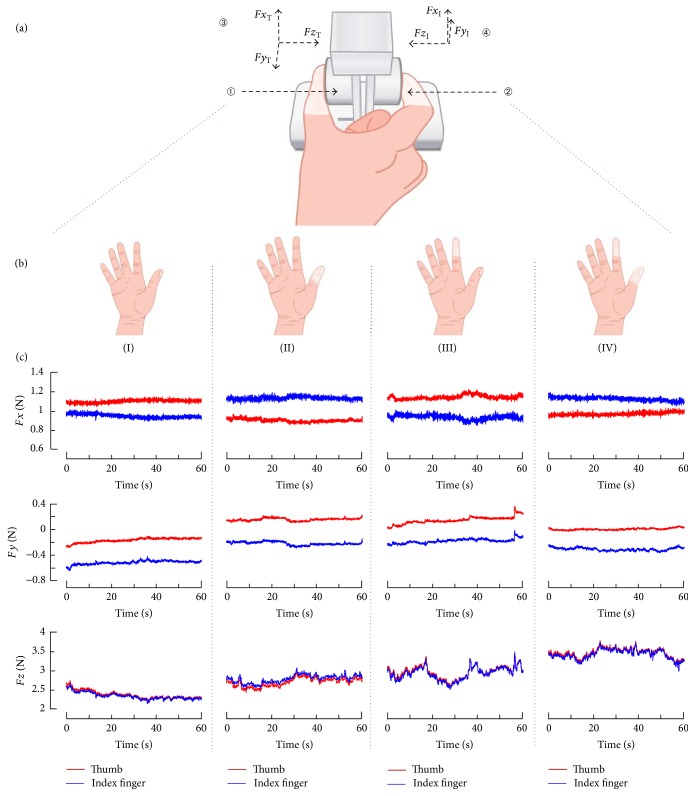
Experimental setup, tactile conditions, and representative data. (a) The apparatus and posture for precision grip; (b) tactile conditions; and (c) force signals of the thumb and index finger from a representative subject.** ①** Force transducers were instrumented inside the shields.** ②** Fingertips were wrapped with polyethylene films.** ③** and** ④** Local coordinate systems for the thumb and index finger, respectively. Tactile conditions I: nondigit was blocked; II: only the thumb was blocked; III: only the index finger was blocked; and IV: both the thumb and index finger were blocked. The curves shown in (c) are the force components of the thumb and index finger in the* x*-,* y*-, and *z*-axis with respect to the local coordinate systems.

An apparatus was designed to measure the forces at digit-object interface (Figure 1(a)). Two miniature 6-component force/torque transducers (Nano17, ATI Industrial Automation, Inc., Apex, NC) were instrumented inside plastic shields for the thumb and index finger, respectively (Figure 1(a)). The resolutions of each transducer was equally 0.0125 N in the *x*-, *y*-, and *z*-axis. The *x*-axis and *y*-axis were along the vertical and horizontal directions in the surface plane of each transducer, and the *z*-axis was in the perpendicular direction to the contact surface (Figure 1(a)). The signals were amplified and multiplexed using a custom ATI interface box (ATI Industrial Automation, Inc., Apex, NC) and converged to a 16-bit analog-digital converter (PCIe-6343, National Instrument, Austin, TX). The force signals were recorded simultaneously at a sampling frequency of 1000 Hz. The pinching surfaces were covered with 100-grit sandpaper and oriented in parallel with a pinch span of 50 mm. The gross weight of the instrumented apparatus was 172 g. Digit force data collection and real-time visual feedback were implemented using a custom Labview program (National Instrument, Austin, TX).

### 2.3. Simulated Tactile Deficits

A simulated tactile perturbation was realized by wrapping up the distal pads of thumb and index finger at the right hand with transparent polyethylene films. The films have an average thickness of 0.01  mm and were tightly wrapped in 7 layers around the fingertip for each subject (Figure 1(b)). There were totally 4 conditions: (I) nondigit was blocked; (II) only the thumb was blocked; (III) only the index finger was blocked; and (IV) both the thumb and index finger were blocked. For each condition, fingertip tactile sensitivity was verified using Semmes–Weinstein monofilament (SWM) test following a standard protocol [13].

### 2.4. Test Procedures

Participants sat in a chair at the testing table, with their right upper arm abducted to 20° in the frontal plane and flexed to 30° in the sagittal plane and with their elbow flexed to approximately 90° in the sagittal plan. Their forearm was strapped on a support and their wrist was in a neutral pronation/supination position. Each subject was asked to grip and hold the apparatus with the tips of the thumb and index finger as stably as possible over 60 s. The middle, ring, and little fingers of the gripping hand were flexed to avoid contacting the force transducers. During holding the apparatus, subjects were instructed to maintain the base of the apparatus horizontally, about 5 cm above the testing table, using a minimum grip force to prevent the apparatus from slipping. In order to avert visual feedback, the subjects were instructed to position their digits closely to the apparatus's gripping surfaces but without touch and then occlude their vision with an opaque sleeping eye mask. Six trials were tested for each tactile condition. The testing order for the four conditions was randomly selected in order to minimize an effect of learning. A 1 min rest was given between two consecutive trials, and a 5 min rest was provided between two tactile conditions, in order to limit a potential effect of muscle fatigue. Each subject was familiarized with the testing protocol before the formal experiment.

### 2.5. Data Analysis

For each trial, the signals at the first few seconds were excluded, leaving the last 60 s of data for the following analysis. The three-directional force components of the thumb and index finger from a representative subject are shown in Figure 1(c). The structure of force variability was examined using detrended fluctuation analysis (DFA) [14]. The algorithm of the DFA has been described in our previous publication [10]. Briefly, the force signal was first detrended, integrated, and then divided into windows of equal length *m* (*m* = 10 to 1000 points). In each window, the local trend was estimated using a linear least-squares fit. The root mean square (RMS) was calculated over the window of length *m*. The slope of the function between the RMS and the window size on a double logarithmic graph was the scaling exponent *α*_DFA_.

The force structural correlation between the two digits was quantified using a detrended cross-correlation analysis (DCCA) [10]. According to the DCCA algorithm, the forces of the thumb and index finger were integrated, and the covariance between the integrated signals was calculated. This covariance was divided into* N*-*m* overlapping windows, where *N* is the number of points of each time series and *m* is the window size. Within each window, a linear least-squares fit was calculated and the covariance of the residuals was computed. The detrended covariance was calculated by averaging the covariance over all windows. The scaling exponent, *α*_DCCA_, is the slope of the linear relationship between the window size and the detrended covariance on a log-log plot. Both *α*_DFA_ and *α*_DCCA_ have similar scales and physical meanings. For example, they both exhibit landmark values at 0.5, 1, and 1.5, indicating the structure of force or that of the force correlation corresponds to white, pink, and brown noise, respectively. Values between 0.5 and 1 indicate persistent long-range power-law correlations, whereas those between 1 and 1.5 denote high correlations without power law [14, 15]. The mean and standard deviation of *α*_DFA_ of each individual digit force and *α*_DCCA_ between the two digits were calculated for the four tactile conditions. *α*_DFA_ and *α*_DCCA_ were implemented in MATLAB (MathWorks, Natick, MA, USA).

### 2.6. Statistical Analyses

Statistical analyses were performed using SigmaStat (Version 3.5, Systat Software Inc., San Jose, CA). Normality of data was tested using the Kolmogorov-Smirnov test. Two-way repeated measures ANOVAs (2 × 2) were performed to evaluate the effects of digit (thumb or index finger) and of the tactile conditions (I, II, III, and IV) on *α*_DFA_. One-way repeated measures ANOVAs were used to examine the effects of tactile conditions on *α*_DCCA_. The Huynh-Feldt correction was used when the assumption of sphericity was violated. Post hoc pairwise comparison was performed using the Holm-Sidak test. Friedman repeated measures analysis of variance on ranks was applied if the normal distribution was not satisfied. A *p* value of less than 0.05 was considered statistically significant.

## 3. Results

The SWM scores of the thumb and index finger under the tactile conditions are demonstrated in Table 1. The scores of the thumb are 3.75 ± 0.32 and 3.79 ± 0.22 under conditions II and IV, respectively, significantly higher than that under condition I (2.62 ± 0.29, *p* < 0.001). Similarly, the scores of the index finger are 3.63 ± 0.39 and 3.66 ± 0.35 under III and IV, respectively, which are significantly higher than the scores under condition I (2.56 ± 0.20, *p* < 0.001). No significant difference was found in the SWM scores between III (2.62 ± 0.29) and I for the thumb (*p* = 0.991), or between II (2.59 ± 0.25) and I for the index finger (*p* = 0.873).

**Figure 2:**
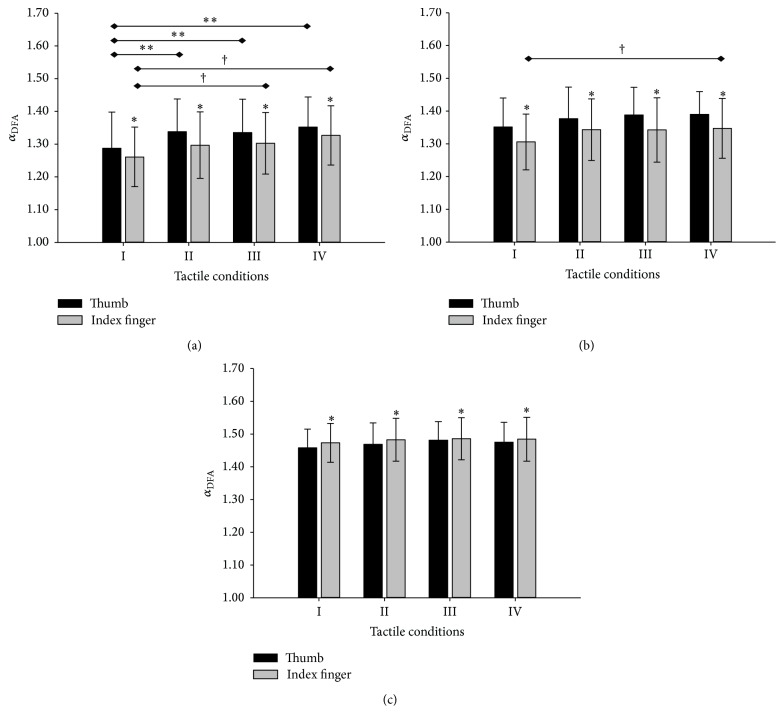
Effects of tactile sensitivity on the structural variability of digit forces. Results are illustrated as the mean and standard deviation of *α*_DFA_ values for (a) *Fx*, (b) *Fy*, and (c) *Fz* of the thumb and index finger. ^*∗*^Significant difference between the two digits (*p* < 0.05). ^*∗∗*^Significant difference between tactile conditions for the thumb (*p* < 0.001). ^†^Significant difference between tactile conditions for the index finger (*p* < 0.05).

The force components in the *x*-, *y*-, and *z*-axis during precision grip from one representative subject are depicted in Figure 1(b). The ANOVA test showed significant main effects of tactile condition on *α*_DFA_ of *Fx* (*F*_3,239_ = 6.814, *p* < 0.001, Figure 2(a)) and that of *Fy* (*F*_3,239_ = 6.814, *p* = 0.032, Figure 2(b)). The pairwise comparison showed that *Fx* of thumb under conditions II, III, and IV had higher *α*_DFA_ values than that under condition I; and *Fx* of the index finger had higher *α*_DFA_ under conditions III and IV than under condition I (Figure 2(a)). A significantly higher *α*_DFA_ value was found in *Fy* of the index finger under condition IV than under condition I (*p* = 0.046, Figure 2(b)). No significant difference was observed in *α*_DFA_ of *Fz* among the four tactile conditions (*p* = 0.412, Figure 2(c)).

The digits significantly affected *α*_DFA_ of *Fx* (*F*_1,239_ = 16.266, *p* < 0.001, Figure 2(a)), *Fy* (*F*_1,239_ = 29.774, *p* < 0.001, Figure 2(b)), and *Fz* (*F*_1,239_ = 14.746, *p* < 0.001, Figure 2(c)). Compared to the thumb, the index finger showed significantly lower *α*_DFA_ of *Fx* (Figure 2(a)) and of *Fy* (Figure 2(b)); but a reverse relation was found in *Fz*, showing that *α*_DFA_ of index finger was significantly higher than that of the thumb (Figure 2(c)).

**Figure 3:**
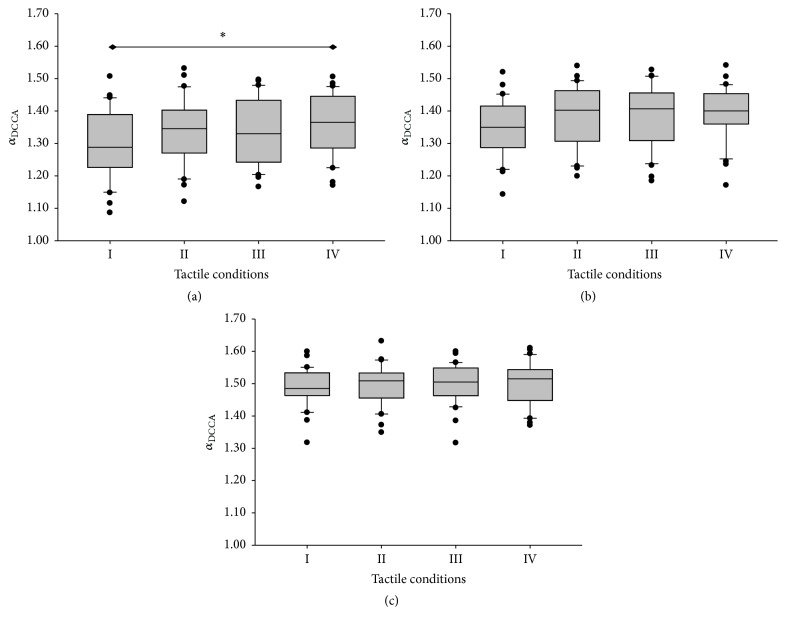
Effects of tactile sensitivity on the structural variability of interdigit force correlation. The box plots depict the 5th, 25th, 50th, 75th, and 95th percentiles and outliers of *α*_DCCA_ for* Fx* (a),* Fy* (b), and* Fz* (c). ^*∗*^Significant difference between the thumb and index finger (*p* < 0.001).

Effects of tactile sensation were found on *α*_DCCA_ of *Fx* (*χ* ^2^ = 16.440, *p* < 0.001, Figure 3(a)). *α*_DCCA_ of *Fx* under IV was significantly higher than under I. No significant difference was found in *α*_DCCA_ of either *Fy* (*p* = 0.051) or *Fz* (*p* = 0.069) among the four tactile conditions (Figures 3(b) and 3(c)).

## 4. Discussion

This study examined the effects of fingertip tactile sensitivity on the structural variability of the thumb and index finger forces during stable precision grip. Results of DFA showed that with reduced tactile sensitivity the shear force components in the vertical direction of the thumb or index finger had lower structural variability (Figure 2(a)). This finding was in line with the previous finding from the effects of visual feedback on digit force regulation during single-digit pressing [16, 17] and two-digit pinching [10, 11]. For example, absence of visuomotor correction can reduce the irregular patterns in digit force structures compared to the condition with visual feedback [10, 11, 16, 17]. The simplified time-dependent structure of the vertical shear forces at the digits with tactile deficits reflected a decreased motor control flexibility in response to external variables.

In contrast to *Fx*, tactile senility had little impact on the structural variability of normal force *Fz* or the horizontal shear force *Fy*, except for *Fy* of the index finger between IV and I (Figures 2(b) and 2(c)). This finding implied that the normal and vertical shear forces during stable precision grip would be controlled by relatively independent processes, which corroborates with the observations from the independent yet highly coupled grip force (the average of digit normal forces) and load force (the sum of digit vertical shear forces) control during object loading, releasing, or point-to-point moving [18, 19]. It has been recognized that both the experience-based feed-forward control and the sensory-driven feedback control can be involved in regulation of grip and load forces [20, 21]. The results of the current study would thus suggest that, in contrast to the vertical shear force whose structures highly relied on tactile feedbacks, the normal force structures are more likely controlled by the feed-forward strategy.

The effects of tactile sensitivity on digit force variability may also have neurophysiologic implications. In the fingertip tactile signal encoding, it is the slow-adapting (SA-I and SA-II) rather than the fast-adapting (FA-I and FA-II) afferent fibers that are most likely excited by the sustained skin deformation and sensitive to the static force applied upon the digits [22–24]. Therefore, it would be the SA afferent fibers that play a leading role in providing tactile information during the stable precision grip. Considering that the SA-II afferent fibers can respond to the lateral stretching of the skins and are “sensitive to the tangential shear strain to the skin that occurs during object manipulation” [22], we thus postulated that the lowered structural variability observed from *Fx* with reduced tactile sensitivity would be attributed to the insensitivity SA-II fibers achieved by the tactile perturbation (Figure 2(a)).

For the normal force, the index finger showed lower structural variability (higher *α*_DFA_) than the thumb (Figure 2(c)), which was consistent with the notion that the index finger is less flexible in control of digit normal force than the thumb [11]. For *Fx*, the index finger showed higher structural variability than the thumb, revealing an interdigit difference in organizing force structures against the load during precision grip (Figures 2(a) and 2(b)). There were no interaction between the tactile condition and digits, which excluded the tactile sensation as a factor making change of the digit-specific force structural control.

Tactile sensitivity affected *α*_DCCA_ of the vertical shear force (Figure 3). In comparison of normal tactile conditions, *α*_DCCA_ of the vertical shear force significantly increased when both the thumb and index finger suffered tactile block. This increased *α*_DCCA_ indicated lowered variability of interdigit shear force correlation, which suggested an intensified coupling of the engaged digits and lowered dynamical degrees of freedom of the motor system in response to the increased uncertainty of the load due to the blurred tactile inputs [10–12, 25]. It should be noted that tactile deficits on single digit did not lead to a change of interdigit shear force correlation (see conditions II and III in Figure 3(a)). It would be postulated that a “cross talk” mechanism exists between the two digits to compensate the lack of tactile information and to maintain suitable interdigit force correlation for stable grip control.

In this study, a transient tactile perturbation was realized by polyethylene that was tightly wrapped around digit tips. Although it provided a fast and effective way to modulate the digit-tip tactile sensitivity and to switch the tactile conditions between I and IV, this method has a limitation that covering digit tips changed the friction of the contact surface, which would potentially interfere with the results. However, by theoretical analysis and experimental verification, we may prove that the friction change due to wrapping up digits has little influence on their force variability control for precision grip; it was the tactile sensation rather than the friction that should be responsible for the findings. For this point, more details are available in the supplementary document (in Supplementary Material available online at http://dx.doi.org/10.1155/2016/8314561).

## 5. Conclusions

Deficits of fingertip tactile sensitivity lowered the structural variability of vertical shear force at each suffered digit and that of interdigit vertical shear force correlation during stable precision grip. The tactile sensitivity did not affect the time-dependent structure of digit normal forces. These results indicated a reduction in the digit force flexibility and an increase in the interdigit coupling in response to the blurred tactile information. This study shed light on different strategies involved in the control of the shear and normal forces and the role of SA-II afferent fibers in regulation of vertical shear force for stable grip.

## Supplementary Material

Systemic recurrence (metastasis) sites were shown in Supplementary Table 1.

## Figures and Tables

**Table 1:** Semmes–Weinstein monofilament test scores under the four tactile conditions.

	Tactile conditions			
	I	II	III	IV
Digits				
Thumb	2.62 ± 0.29	3.75 ± 0.32^*∗*^	2.62 ± 0.29	3.79 ± 0.22^*∗*^
Index finger	2.56 ± 0.20	2.59 ± 0.25	3.63 ± 0.39^*∗*^	3.66 ± 0.35^*∗*^

Scores were demonstrated as mean ± standard deviations.

^*∗*^Significantly different from baseline condition I (*p* < 0.001).
