# Supplementary material for: Effects of Tactile Sensitivity on Structural Variability of Digit Forces during Stable Precision Grip
Source: Biomed Res Int. 2016 Oct 25;2016:8314561. doi: 10.1155/2016/8314561 (PMC5099480; doi:10.1155/2016/8314561)
Supplement: Supplementary file 1 — Systemic recurrence (metastasis) sites were shown in Supplementary Table 1. [file 8314561.f1.pdf]

Supplementary Materials

Effects of Tactile Sensitivity on Structural Variability of Digit Forces During Stable Precision Grip

Ke Li[1,*], Na Wei[2], Shouwei Yue[3,*]

[1] Laboratory of Motor Control and Rehabilitation, Institute of Biomedical Engineering, School of Control Science and Engineering, Shandong University

[2] Department of Geriatrics, Qilu Hospital, Shandong University

[3] Department of Physical Medicine and Rehabilitation, Qilu Hospital, Shandong University

*** Correspondence:**

Ke Li, Ph.D, Associate Professor

Laboratory of Motor Control and Rehabilitation, Institute of Biomedical Engineering, School of Control Science and Engineering, Shandong University.

17923 Jingshi Aveue, Jinan, 250061, Shandong, China.

Email: kli@sdu.edu.cn;

Shouwei Yue, M.D, Professor

Department of Physical Medicine and Rehabilitation, Qilu Hospital, Shandong University.

107 Wenhuaxi Road, Jinan, 250012, Shandong, China

Email: shouweiy@sdu.edu.cn

In this study, a tactile perturbation was realized by tightly wrapping up digit tips with polyethylene. It is a fast and effective way to reduce the digit-tip tactile sensitivity and to switch the tactile conditions between I and IV (see the manuscript). The tactile perturbation approach evolves from the method using gloves to prevent the grasping digits from the object's contact surface (Kinoshita 1999). A limitation of this method was that the friction of the contact surface changes with polyethylene, which potentially interfered with the control of digit force variability. In this supplementary document, we will prove, by theoretical analysis and experimental verification, that the friction change due to the specific tactile perturbation approach -- wrapping up digits -- has little influence on the force variability control during precision grip; and that it was the tactile sensation rather than the friction that should be responsible for the findings.

First, tactile sensation should be evoked by the cutaneous mechanoreceptors innervating the hairless area of fingertips. To stimulate the mechanoreceptors, the surface of the digit pad needs to be deformed by a certain amount of force. There is a nonlinear relationship between the force and digit tip deformation. This relationship may also describe how the cutaneous mechanorecptors fire with the contact force. According to this relationship, the contact area and fingertip deformation increase extensively as the force changes from 0-3 N but maintain relatively stable with greater force (Jones and Lederman 2006). According to this relationship, if the digit tip surface is preset a certain deformation ahead of grip, then with increasing grip force the deformation will not be aggravated, and little additional tactile information will be gained during grip (Westling and Johansson 1987, Serina, Mockensturm et al. 1998, Jones and Lederman 2006).

The role of the tightly wrapped polyethylene in our study was to generate a presetting deformation of digit surface (Figure S1 (c)), which could extensively restricted the function of cutaneous mechanoreceptors during grip. This is a key difference from wearing gloves, which

simply blocks the direct contact between the digit and object ((Figure S1 (b)). With the tightly wrapped polyethylene for precision grip, the friction information at the contact interface, as well as the other tactile information, would be no longer detected by the mechanoreceptors, and thus have little influence on the digit force control.

Second, in psychophysics the haptic perception on the friction condition (e.g. roughness and smoothness) relies on tangential relative motion or sliding in the contact surface. Wearing gloves cannot stop the relative motion or sliding between the digit tips and the glove. Therefore the material properties of the gloves, e.g. the rubber's coefficient of friction, should be account for the haptic perception. In our study, the polyethylene was tightly wrapped around the digit pads, which could minimize the shear-directional relative motion or sliding.

Third, in this study we analyzed the time-dependent structural variability of digit forces using detrended fluctuation analysis (DFA). Different from the force vector directions that are sensitive to the friction condition (Seo and Armstrong 2009), the structural variability quantified by DFA is associated with sensorimotor process and relatively robust to the environmental noise (Li, Marquardt et al. 2013, Li, Evans et al. 2015, Li, Wei et al. 2015). The algorithm we used in this study could further limit the influence of intended friction change during precision grip.

Above we have elucidated why we judged that friction would not be a major factor influencing the digit force variability performance in our study. In order to examine our judgement, here we designed an additional experiment (Figure S2). Two friction conditions will be compared: (A) the digit pads were tightly wound with polyethylene as exactly what we did in the manuscript (Figure S2 (A)); and (B) the digit tips was applied with lubricating oil before wrapped with polyethylene (Figure S2 (B)). The hypothesis is that, if the friction affects

the testing results, the force structural variability under condition (A) and condition (B) should be different.

Three students who were innocent to the testing purpose participated in this additional experiment. For each tactile condition (I, II, III and IV), each subject performed four grip trials following the exact same protocol as we did in the previous experiment. The DFA and DCCA were applied to analyze the structural variability of digit forces during precision grip. Before the grip test, the tactile sensitivity under (A) and (B) was tested using SWM scores.

Table S1 showed that the thumb and index finger with and without lubricating oil had comparable tactile sensitivity. If a significant difference could be found between the two friction conditions (A vs. B), then we could infer that the friction would be a factor influencing the digit force control. Otherwise, the friction would not affect the results.

Figure S3-S5 were the results of DFA calculated from the vertical shear force (Fx), horizontal shear force (Fy), and normal direction force (Fz) under the two friction conditions: A-tactile block without the lubricating oil; and B-tactile block with the lubricating oil. There was no significant difference between the two friction conditions (ANOVA test, $p > 0.05$) for any of the force components (Fx, Fy, Fz), at either the thumb and index finger, or under any of the tactile conditions (I-IV, Figure S4-S6). These results showed that the different coefficient of friction would not lead to a change of DFA values of digit forces. The difference of digit force structural variability among the four tactile conditions was most likely attributed to the altered tactile sensitivity rather than the intended friction conditions.

The DCCA results calculated between the thumb and index finger force components, including the vertical shear force (Fx), the horizontal shear force (Fy) and the normal force (Fz) with and without the lubrication oil, were shown in Figure S6. Similar to the results of DFA, no significant difference was observed in the DCCA between the two friction conditions

(ANOVA test, $p > 0.05$, A and B) for any force components (Fx, Fy, Fz) or under any tactile conditions (I-IV, Figure S6). These results showed that the change of friction did not result in the change of inter-digit force coordination.

In conclusion, in this study the digit tips were tightly wrapped up with polyethylene in order to realize a transient but effective tactile perturbation. Although the friction of the interface between the digit tips and the contact surface changed due to the polyethylene coverage, this friction change had little influence on the results of digit force variability during precision grip. It was the tactile sensation rather than the friction that should be responsible for the findings.

References:

Jones, L. A. and S. J. Lederman (2006). Human Hand Function. Oxford New York, Oxford University Press.

Kinoshita, H. (1999). "Effect of gloves on prehensile forces during lifting and holding tasks." Ergonomics **42**(10): 1372-1385.

Li, K., P. J. Evans, W. H. Seitz, Jr. and Z. M. Li (2015). "Carpal tunnel syndrome impairs sustained precision pinch performance." Clin Neurophysiol **126**(1): 194-201.

Li, K., T. L. Marquardt and Z. M. Li (2013). "Removal of visual feedback lowers structural variability of inter-digit force coordination during sustained precision pinch." Neurosci Lett **545**: 1-5.

Li, K., N. Wei, S. Yue, D. Thewlis, F. Fraysse, M. Immink and R. Eston (2015). "Coordination of digit force variability during dominant and non-dominant sustained precision pinch." Exp Brain Res **233**(7): 2053-2060.

Seo, N. J. and T. J. Armstrong (2009). "Friction coefficients in a longitudinal direction between the finger pad and selected materials for different normal forces and curvatures." Ergonomics **52**(5): 609-616.

Serina, E. R., E. Mockensturm, C. D. Mote, Jr. and D. Rempel (1998). "A structural model of the forced compression of the fingertip pulp." J Biomech **31**(7): 639-646.

Westling, G. and R. S. Johansson (1987). "Responses in glabrous skin mechanoreceptors during precision grip in humans." Exp Brain Res **66**(1): 128-140.

Figure Legends:

Figure S1: Tactile block with polyethylene. (A) Fingertip without applied force; (B) fingertip deformation by contact forces; (C) Preset deformation by tightly wrapping polyethylene around the digit tips.

Figure S2: Schema of the experiment design. (A) Digits were wrapped up by polyethylene following the previous experiment in manuscript; (B) Change interface friction by applying digit tips with lubricating oil.

Figure S3: DFA of the vertical shear forces (Fx) at the thumb and index finger with different friction conditions: (A) without lubricating oil, and (B) with lubricating oil.

Figure S4: DFA of the vertical shear forces (Fy) at the thumb and index finger with different friction conditions: (A) without lubricating oil, and (B) with lubricating oil.

Figure S5: DFA of the vertical shear forces (Fz) at the thumb and index finger with different friction conditions: (A) without lubricating oil, and (B) with lubricating oil.

Figure S6: DCCA of the Fx, Fy and Fz with different friction conditions: (A) without lubricating oil, and (B) with lubricating oil.

Figure S1:

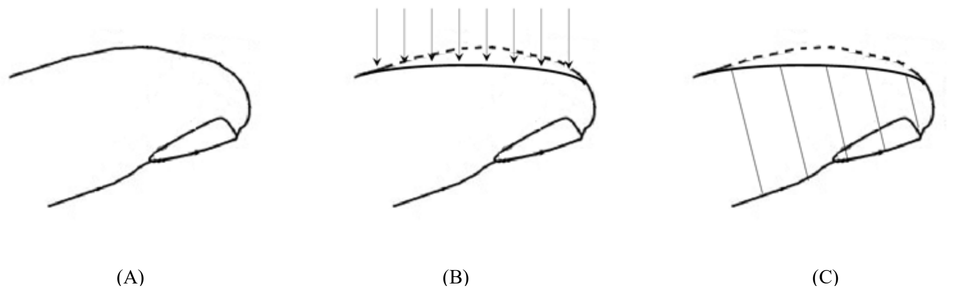

Figure S2:

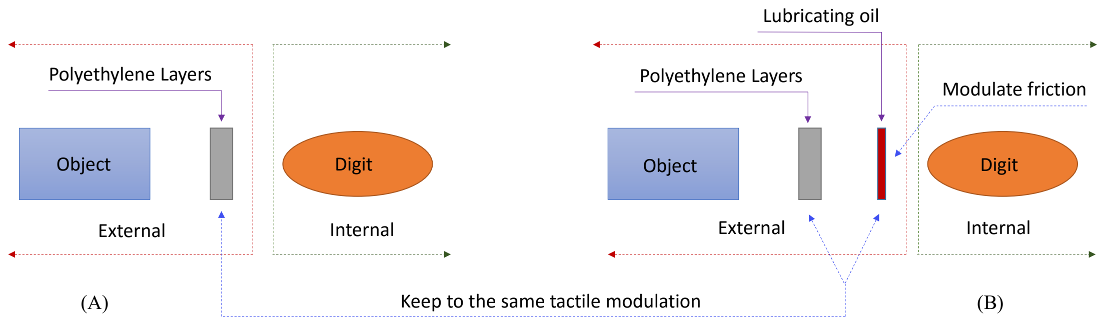

Figure S3:

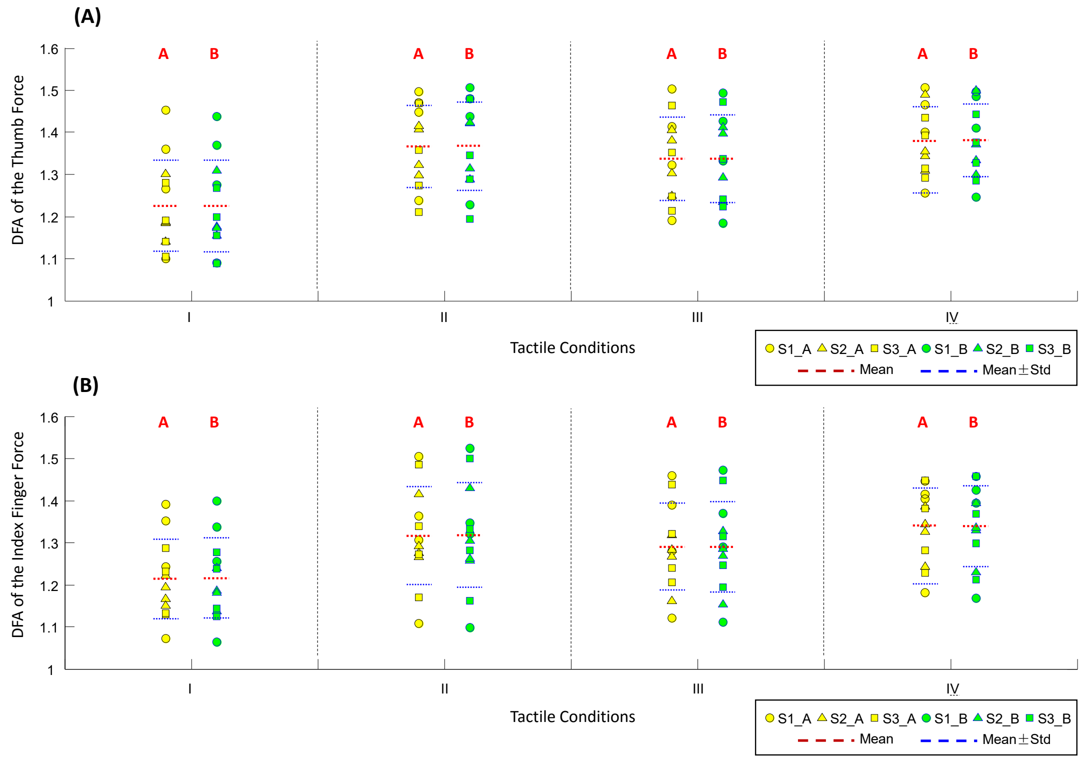

Figure S4:

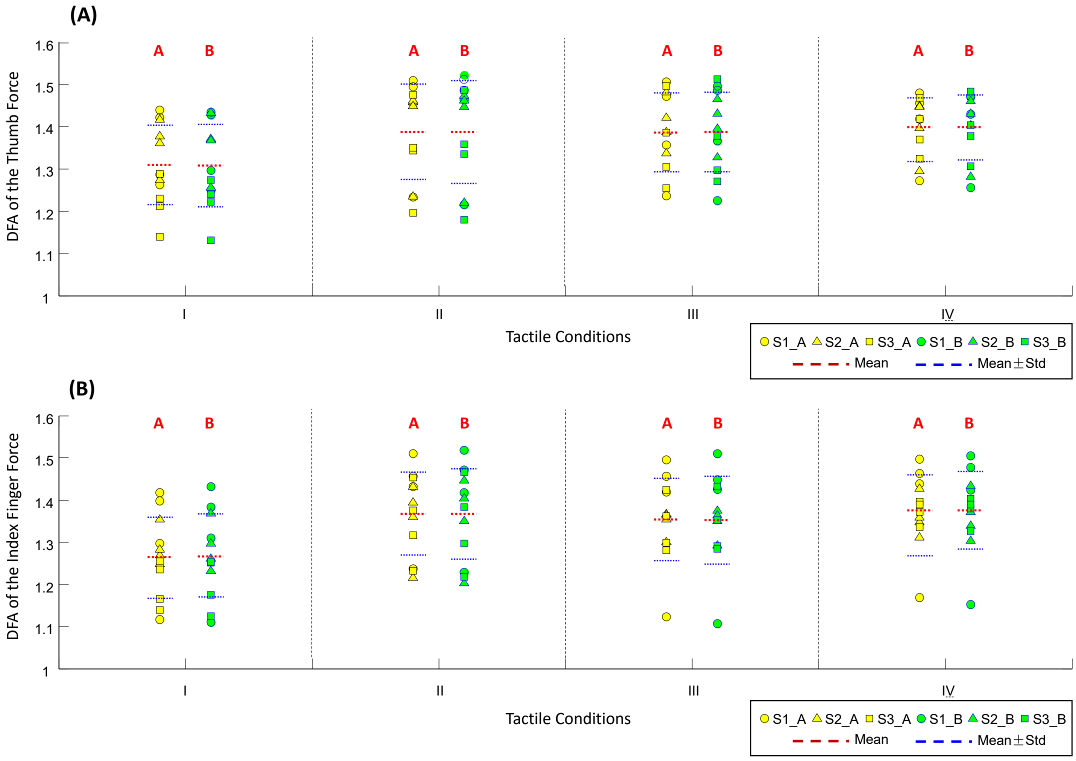

Figure S5:

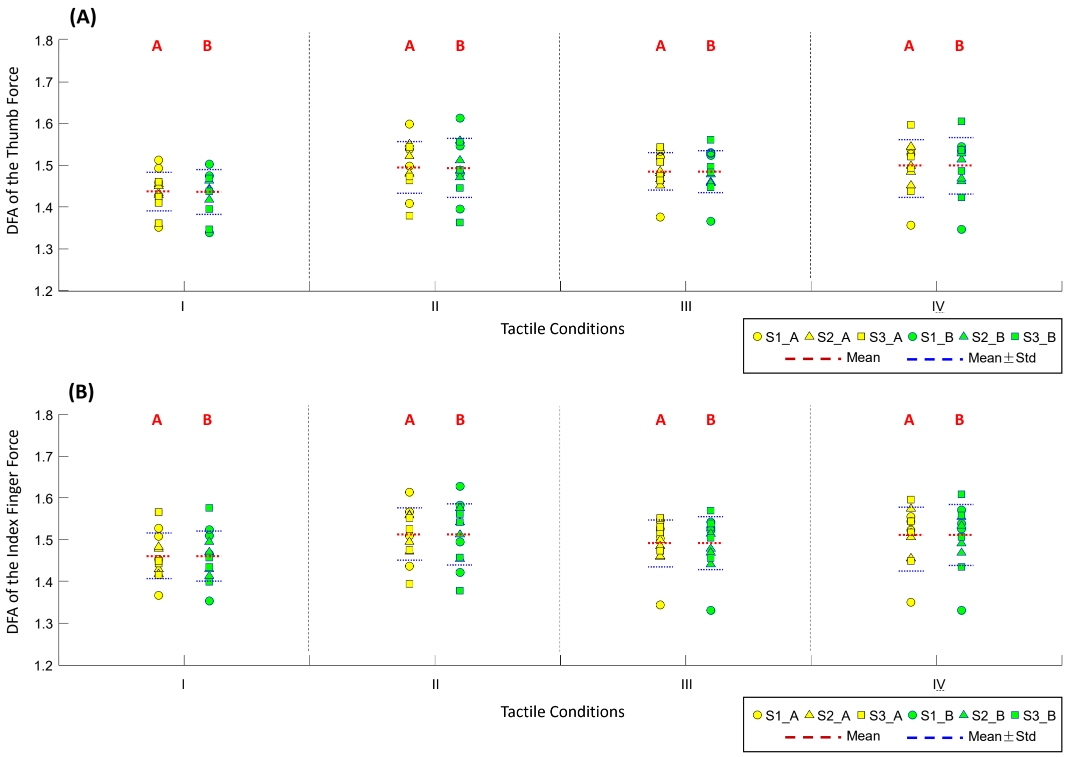

Figure S6:

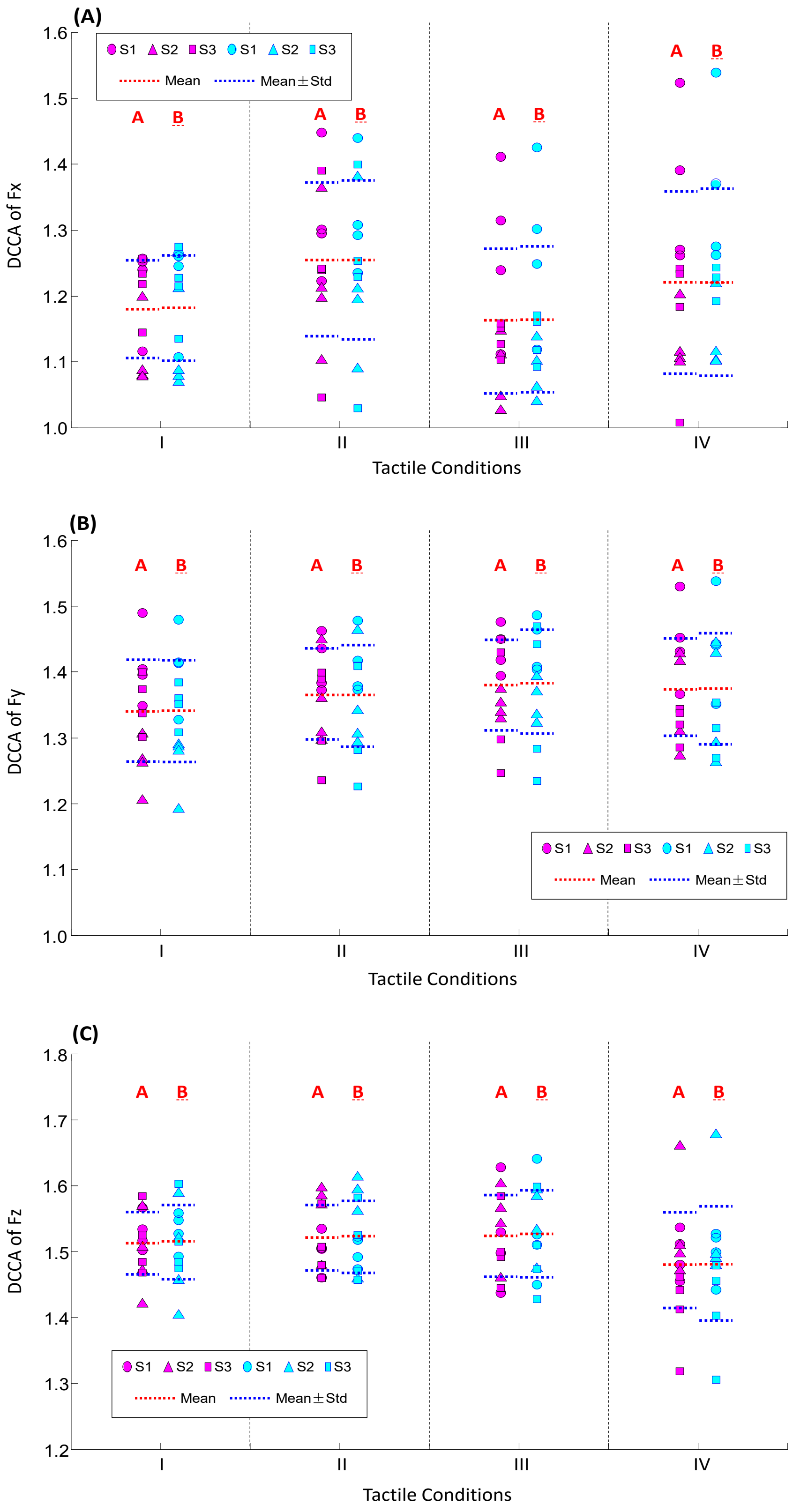
(A)
S1 S2 S3 S1 S2 S3
Mean
Mean±Std
A B
DCCA of Fx
1.6
1.5
1.4
1.3
1.2
1.1
1.0
I
II
III
IV
Tactile Conditions
(B)
DCCA of Fy
1.6
1.5
1.4
1.3
1.2
1.1
1.0
Tactile Conditions
(C)
DCCA of Fz
1.8
1.7
1.6
1.5
1.4
1.3
1.2
Tactile Conditions

Table:

Table S1. SWM scores with different coefficient of friction

No	Cond	I		II		III		IV	
		Thumb	Index	Thumb	Index	Thumb	Index	Thumb	Index
S1	A	2.44	2.44	3.84	2.44	2.44	2.83	3.84	3.84
	B	2.44	2.44	3.84	2.44	2.44	2.83	3.84	3.84
S2	A	2.44	2.44	3.84	2.44	2.44	3.22	4.08	3.84
	B	2.44	2.44	3.61	2.44	2.44	3.61	3.84	3.84
S3	A	2.44	2.44	3.61	2.44	3.22	3.84	3.84	3.61
	B	2.44	2.44	3.84	2.44	3.22	3.61	3.84	3.84

S1-S3 were the three students who participated in the supplementary test. I-IV were the four tactile conditions; A and B were the two friction conditions.
